# Epitope-Based Vaccines with the *Anaplasma marginale* MSP1a Functional Motif Induce a Balanced Humoral and Cellular Immune Response in Mice

**DOI:** 10.1371/journal.pone.0060311

**Published:** 2013-04-08

**Authors:** Paula S. Santos, Angela A. S. Sena, Rafael Nascimento, Thaise G. Araújo, Mirian M. Mendes, João R. S. Martins, Tiago W. P. Mineo, José R. Mineo, Luiz R. Goulart

**Affiliations:** 1 Laboratory of Nanobiotechnology, Institute of Genetics and Biochemistry, Federal University of Uberlândia, Uberlândia, MG, Brazil; 2 Laboratory of Protein Chemistry and Natural Products, Institute of Genetics and Biochemistry, Federal University of Uberlândia, Uberlândia, MG, Brazil; 3 Laboratory of Parasitology, Institute of Veterinary Research Desidério Finamor, Eldorado do Sul, RS, Brazil; 4 Laboratory of Immunoparasitology, Institute of Biomedical Sciences, Federal University of Uberlândia, Uberlândia, MG, Brazil; 5 Department of Medical Microbiology and Immunology, School of Medicine, University of California Davis, Davis, California, United States of America; Instituto Butantan, Brazil

## Abstract

Bovine anaplasmosis is a hemoparasitic disease that causes considerable economic loss to the dairy and beef industries. Cattle immunized with the *Anaplasma marginale* MSP1 outer membrane protein complex presents a protective humoral immune response; however, its efficacy is variable. Immunodominant epitopes seem to be a key-limiting factor for the adaptive immunity. We have successfully demonstrated that critical motifs of the MSP1a functional epitope are essential for antibody recognition of infected animal sera, but its protective immunity is yet to be tested. We have evaluated two synthetic vaccine formulations against *A. marginale*, using epitope-based approach in mice. Mice infection with bovine anaplasmosis was demonstrated by qPCR analysis of erythrocytes after 15-day exposure. A proof-of-concept was obtained in this murine model, in which peptides conjugated to bovine serum albumin were used for immunization in three 15-day intervals by intraperitoneal injections before challenging with live bacteria. Blood samples were analyzed for the presence of specific IgG2a and IgG1 antibodies, as well as for the rickettsemia analysis. A panel containing the cytokines’ transcriptional profile for innate and adaptive immune responses was carried out through qPCR. Immunized BALB/c mice challenged with *A. marginale* presented stable body weight, reduced number of infected erythrocytes, and no mortality; and among control groups mortality rates ranged from 15% to 29%. Additionally, vaccines have significantly induced higher IgG2a than IgG1 response, followed by increased expression of pro-inflammatory cytokines. This is a successful demonstration of epitope-based vaccines, and protection against anaplasmosis may be associated with elicitation of effector functions of humoral and cellular immune responses in murine model.

## Introduction

Bovine Anaplasmosis manifested as a severe hemolytic disease is caused by an obligate intraerythrocytic bacterium, the *Anaplasma marginale*, endemic in tropical and subtropical regions, which can be transmitted biologically by ticks and mechanically by blood-contaminated fomites or biting flies [1]. It results in considerable economic loss mainly due to the low weight gain, reduction in milk production, abortion, treatment costs, and mortality [2].

Several immunization models have been successfully used to determine the usefulness of novel antigens and strategies for vaccination, and most of the immunization protocols against *A. marginale* include several recombinant major surface proteins (MSPs) and plasmids [3]–[7]. Although the efficacy of these experimental vaccines is variable between and within experiments, they demonstrate the feasibility of a subunit vaccine approach for the establishment of immunization protocols against this disease. One potential class of targets for development of such a subunit vaccine would be functional factors associated with adhesion to and invasion of bovine erythrocytes [8].

*A. marginale* MSPs are involved in interactions with both vertebrate and invertebrate hosts [9]–[11]. Immunity against *A. marginale* is associated with these proteins, which are exposed in the rickettsia surface, are easily accessible by the host immune system, and can be neutralized by antibodies against exposed epitopes [12], [13]. Cattle immunized with *A. marginale* outer membrane proteins developed high antibody titers and presented significant rickettsemia reduction if challenging with the pathogen when compared to adjuvant-immunized controls [14].

The MSP1 is a heteromeric complex of a single MSP1a protein covalently associated with MSP1b polypeptides [15], and the MSP1a has been shown to be involved in adhesion of *A. marginale* to host cells [10], [11], [16], and possess a conserved neutralization-sensitive epitope [17]. Cattle immunized with MSP1 presented protective humoral immune response [14], and this specific response was preferentially directed to the carboxyl-terminal region of MSP1a, which stimulated high levels of IFN-Υ production by CD4+ T cells [15]. This cytokine activates macrophages and increases nitric oxide production that are effector molecules against rickettsia [18]; moreover, IFN-Υ acts on B cells by stimulating the IgG_2_ production [19].

Vaccines are usually based on the native immunogen or on whole recombinant antigens, but responses are not always optimal and efficacy is variable, probably because of unequal or incorrect exposure of critical epitopes. Therefore, the immunodominance of epitopes seems to be a key factor limiting the type and breadth of adaptive immunity [20]. Advances in understanding the mechanisms of immunodominance, represent an opportunity to further develop an epitope-based approach. Generally, protection induced by natural immunogens not always reaches the optimal response due to cross-reactions or to unstable exposure of the epitope; however, it is hypothesized that improvements can be achieved by identifying specific motifs that may enhance this response. Recently, we have demonstrated that a critical motif, STSSxL, is essential for antibody recognition of infected animal sera, which was mapped to the carboxy-terminal end of the MSP1a 28-amino acid functional epitope sequence [21].

In this work, we have evaluated the ability of critical motif sequences in inducing a protective immune response against *A. marginale* in a murine model.

## Materials and Methods

### Bacteria, Antigens and Synthetic Peptides

The *A. marginale* isolate was obtained from blood samples taken for routine diagnostic purposes from naturally infected cattle housed at the Parasitology Unit of the Instituto de Pesquisas Veterinárias Desidério Finamor, Eldorado do Sul, Rio Grande do Sul State (RS), Brazil, and diagnosis was confirmed by PCR and microscopy analyses of the blood smear (parasitemia >30% of red blood cells). The *A. marginale* lysate antigen (ALA) was prepared by subjecting *A. marginale*-infected erythrocytes to six cycles of freeze-thaw lysis.

The peptides sequences were constructed based on the motifs STSSQL (Am1) and SEASTSSQLGA (Am2) as previously described [21]. Both peptides were chemically synthesized (GenScript USA Inc.) with 26 residues, and coupled to Bovine Serum Albumin to increase immunogenicity. Am1 contains three repeats of the motif sequence (STSSQLGGGSSTSSQLGGGSSTSSQL), whereas Am2 contains two repeats (SEASTSSQLGAGGGSSEASTSSQLGA), both separated by a 4-aa linker (GGGS).

### Mice

All experimental procedures were conducted in accordance with the ethical principles of the Brazilian Academy of Animal Experimentation and were approved by the Animal Research Ethics Committee of the Federal University of Uberlandia under the protocol number 017/11. The experiments were carried out with 4–6 week old female BALB/c mice.

### Quantitative PCR of *A. marginale*-infected Mice

To investigate mice erythrocytes infection with *A. marginale*, ten animals were submitted to intraperitoneal injections of 3×10^5^ rickettsia. All animals were euthanized 30 days after inoculation, and blood samples were collected on days 0, 15 and 30 for blood smears analyses. An additional 50-µl volume for each sample was used for genomic DNA extraction, as described elsewhere [22].

Primers targeting the MSP5 gene of *A. marginale* (Forward: 5′-TCAGATGCTCACAGGCGAAG-3′; Reverse: 5′-CGACATACCTGCCTTTCCCA-3′) were designed using the Primer Express 3.0 Program (PE Applied Biosystems, CA).

Standards curves were constructed by cloning PCR products of MSP5 gene fragments using TOPO TA Cloning Dual Promoter Kit (Life Technologies), and then transformed into chemically competent *E.coli* DH10B competent cells (ElectroMAX DH10B™ Cells - Life Technologies). Plasmids were extracted using a QIAprep spin miniprep kit (Qiagen) and sequenced using MegaBACE 1000 automatic sequencer (Molecular Dynamics). The sequencing reaction was carried out using the DyEnamic ET Dye Terminator Cycle Sequencing Kit (GE Healthcare) following the manufacturer’s instructions. The recombinant plasmid DNA was linearized with the restriction enzyme FastDigest EcoRI (Fermentas). The number of copies was calculated, and standard serial dilutions were made in the range of 10^5^ to 10^8^ copies per µL of the MSP5.

The qPCR assay was carried out in a 7300 Real-time PCR System (PE Applied Biosystems, CA). For absolute quantifications of *A. marginale* MSP5 gene copy numbers, 2 µL of extracted genomic DNA was transferred to 5 µL of PCR SYBR Green qPCR Master Mix reagent and 0.5 pmol (each) primers. Thermal cycling was conducted using the Universal Program profile (PE Applied Biosystems, CA). Each reaction was run in duplicates for quality assurance and statistical analysis purposes. To determine the specificity of amplification, a melting curve analysis was performed after the last cycle of amplification. Once the bacterial copy number was determined for the DNA template, the number of organisms per µl of whole blood was calculated.

### Immunizations and Challenge

Mice were divided into five groups of 10 mice each. Immunizations were performed with three intraperitoneal injections at 15-day intervals with 10 µg of synthetic peptides Am1 (Am1 group) and Am2 (Am2 group); 10 µg of the ALA (ALA group); Freund’s adjuvant (FrA group), and diluent only (PBS group). All peptides were emulsified with complete Freund’s adjuvant for the first immunization and with incomplete Freund’s adjuvant in subsequent immunizations. Blood samples were collected at 0, 15, 30 and 45 days after immunization, and the sera analyzed for the presence of specific antibodies.

Two weeks after the last immunization, three mice in each group were euthanized and their spleens were removed aseptically and stored at −80°C for RNA extraction and analysis by real time polymerase chain reaction (RT-PCR). The remaining animals were challenged by intraperitoneal injections with 3×10^5^ rickettsia. Negative controls included non-immunized and unchallenged mice (n = 2). Animals were observed daily for mortality and body weight changes. All surviving animals were euthanized at 30 days after challenge for analysis of protection against challenge.

In order to determine the peptides’ toxicity used in vaccine formulations, MTT cell viability assays (3-(4,5-dimethylthiazolyl-2)-2,5-diphenyltetrazolium bromide) using the mouse macrophage-like cell line J774A.1 (Sigma-Aldrich) were carried out with Am1, Am2 and PBS treatments. Cells were cultured in RPMI 1640 medium supplemented with 10% fetal bovine serum (FBS) and 0.1% gentamicin (GIBCO, 10 mg/mL) in 5% CO_2_ atmosphere at 37°C. After reaching 80% confluence cells were removed from ﬂasks with trypsin, and resuspended in complete medium prior to centrifugation at 1000 rpm for 5 min. J774.A1 cells were plated on a 96 well plates at a density of 1×10^5^ cells/well and cultured in RPMI culture medium for 4 h to adhere. The cells were cultured for 24 at 37 °C, 5% CO_2_ with peptides in the same concentration used in the vaccine formulation. After each time of incubation 50 µL of MTT solution (5 mg/mL) was added. Cells were re-incubated for 4 h. After this time 50 µL of a solution containing 20% SDS and 50% N,N- dimethyl formamide (pH 4.7) was added and incubated overnight. The amount of viable cells in each well was determined by the absorbance of solubilized formazan. Absorbance was measured in a wavelength of 570 nm (Thermo Plate, TP-Reader). Cell survival rates did not differ from the PBS control (data not shown).

### Detection of Anti-*A. marginale* IgG Production

Optimal assays were established using check board titrations with dilutions of sera, antigen, and conjugates. A ninety-six-well Maxisorp™ microtiter plate (NUNC, NY) was coated with 1 µg/well of each Am1, Am2, and ALA. BSA was used as a control. After overnight incubation at 4°C, the reaction was blocked with 5% BSA in PBS, and serum samples were diluted 1∶50 in blocking buffer and incubated at 37°C for 1 h for IgG, and for 2 h for IgG1 and IgG2a quantifications. After washing, peroxidase-labeled goat anti-mouse IgG (Sigma-Aldrich) or biotinylated anti-mouse IgG1 or anti-mouse IgG2a antibodies (Caltag Lab. Inc., CA), diluted 1∶5000, were added and incubated for 1 h at 37°C. The wells were washed followed by streptavidin-peroxidase (1∶1000; Sigma) incubation. The assays were revealed with OPD Sigma*Fast*™ (Sigma-Aldrich) and read at 492 nm.

ALA was submitted to electrophoresis in a 12% SDS-PAGE [23], and the proteins were transferred as previously described [24]. Immunoblot assays were carried out to verify the *A. marginale* reactivity profile exhibited by mice sera from all animals of each group, at 45 days after immunization. Nitrocellulose strips were blocked with 5% skim milk in PBS, incubated with mouse sera diluted 1∶100. Peroxidase-goat anti-mouse IgG (diluted 1∶5000; Sigma-Aldrich) was used as the secondary antibody. The reaction was developed by adding 0.03% H_2_O_2_ and 3,3′-diaminobenzidine tetrahydrochloride (DAB; Sigma-Aldrich).

### Cytokine Expression by Quantitative Real-time PCR (qPCR)

Total RNA was extracted from the spleen of all challenged mice using TRIzol® reagent according to the manufacturer’s instructions (Invitrogen, USA). RNA samples (2 µg) were reverse-transcribed using MMLV-RT (Amersham Biosciences). To check the appropriated endogenous gene, both GAPDH and β-actin were tested (Table 1). Then, according to the most homogenous amplification of the samples, all PCR amplifications were performed using GAPDH primers to detect the quality of the cDNA.

**Table 1:** Primer sequences for murine cytokines and housekeeping genes used for quantitative RT-PCR.

Genes	Primer Sequence (5′–3′)	Anneling Temp. (°C)	Length (pb)	GenBank accession No.
IL-10	FW: GCCAGGTGAAGACTTTCTTTCAA	60	96	NC_000067.5
	R: TGGCAACCCAAGTAACCCTT			
IL-12	FW: GCATGTGTCAATCACGCTACCT	58	153	NC_000069.5
	R: CCGTCTTCACCATGTCATCTGT			
IL-18	FW: GCATCAGGACAAAGAAAGCCG	60	160	NC_000075
	R: AGTTGTCTGATTCCAGGTCTCCAT			
IFN-γ	FW: TGGAGGAACTGGCAAAAGGAT	56	102	NC_000076.5
	R: GATGGCCTGATTGTCTTTCAAGA			
TGF-β	FW: GAGCCCGAAGCGGACTACT	58	85	NC_000073
	R: CTTTGGTTTTCTCATAGATGGCGT			
TNF-α	FW: GCCCAGACCCTCACACTCAGAT	62	154	NC_000083
	R: GGTTGTCTTTGAGATCCATGCC			
GAPDH	FW: GAAGGTCGGTGTGAACGGATT	58	152	NC_000072.5
	R: TGCCGTGAGTGGAGTCATACTG			

FW, forward primer; R, reverse primer.

To determine the cellular expression of each cytokine, quantitative RT-PCR analysis was performed using the 7300 Real Time PCR Systems (PE Applied Biosystem, CA) and SybrGreen PCR Core Reagent (PE Applied Biosystems, CA). PCR Primers (Table 1) were designed using the Primer Express 3.0 Program (PE Applied Biosystems, CA). The thermal cycling profile used was the Universal Program (PE Applied Biosystems, CA). The change in the expression in the splenocytes samples from Am1, Am2, ALA and FrA were determined by comparing with the data from immunized PBS-control.

### Infected Erythrocytes Visualization by Light Microscopy

Blood samples were taken through cardiac puncture and thin blood smears were prepared immediately after mice euthanasia. Slides were air-dried, fixed in methanol, Giemsa stained and analyzed for the presence of *A. marginale* in the erythrocytes at 100× magnification. 10 isolated fields were examined in each slide, in order to estimate the percentage of infected erythrocytes (PIE), as described elsewhere [25] with some modifications.

### Statistical Analysis

The Kaplan–Meier method was applied to estimate the survival percentage at each time point after challenge and survival curves were compared using the logRank test. Differences between groups were analyzed using the ANOVA test, and the Bonferroni’s multiple comparison post-test was applied to examine all possible pairwise comparisons. Student t test was used for comparison of IgG isotypes and cytokine levels in different groups. Statistical analysis was carried out using GraphPad Prism 5.0 (GraphPad Software Inc., San Diego, CA). A value of p<0.05 was considered statistically significant.

## Results

### *A. marginale* Establishes Infection in Mice

**Figure 1:**
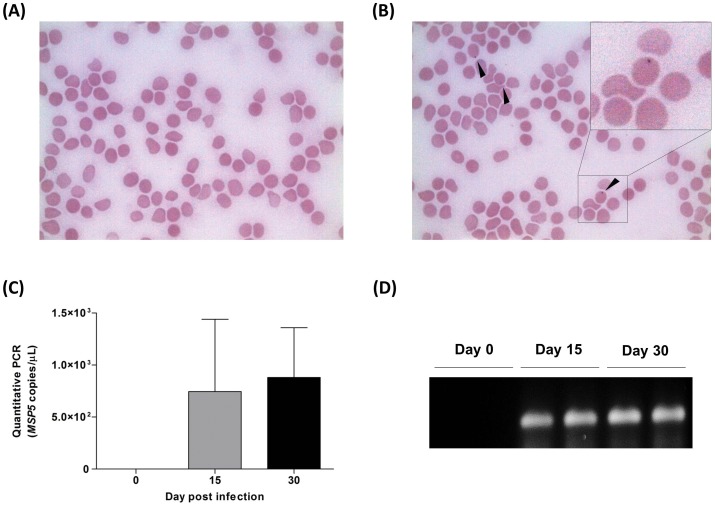
*A. marginale* infection in mice determined by microscopy and qPCR of infected erythrocytes. Mice erythrocytes free of infection (A) were submitted to *A. marginale* injection (3×10^5^ rickettsia) and blood smears were visualized after 30 days (B). MSP5 *A. marginale* copies of infected erythrocytes per µL of blood was quantified by qPCR on days 0, 15 and 30 post-infection (C). The 143-pb MSP5 amplicon is shown in agarose gel electrophoresis (D).

To evaluate the capacity of *A. marginale* infect mice, rickettsia inoculation was performed in 10 animals. Analysis of blood smears revealed the presence of erythrocytes infected with the parasite (Figure 1A and 1B).

Quantitative real time PCR was used to determine the parasitemia load of infected erythrocytes (Figure 1C and 1D). Detection and quantification of MSP5 was observed only after 15 days post-inoculation, with an average of 746 and 881 copies/µL at 15-day and 30-day post-injection, respectively (P>0.05). Amplification efficiency was tested by standard curves for *A. marginale* MSP5 (R^2^ = 0.9986) generated by plotting the value of CT cycle vs. the log of plasmid concentration (from 10^5^ to 10^8^ copies).

### Synthetic Peptides Protect Against *A. marginale* Erythrocyte Infection

**Figure 2:**
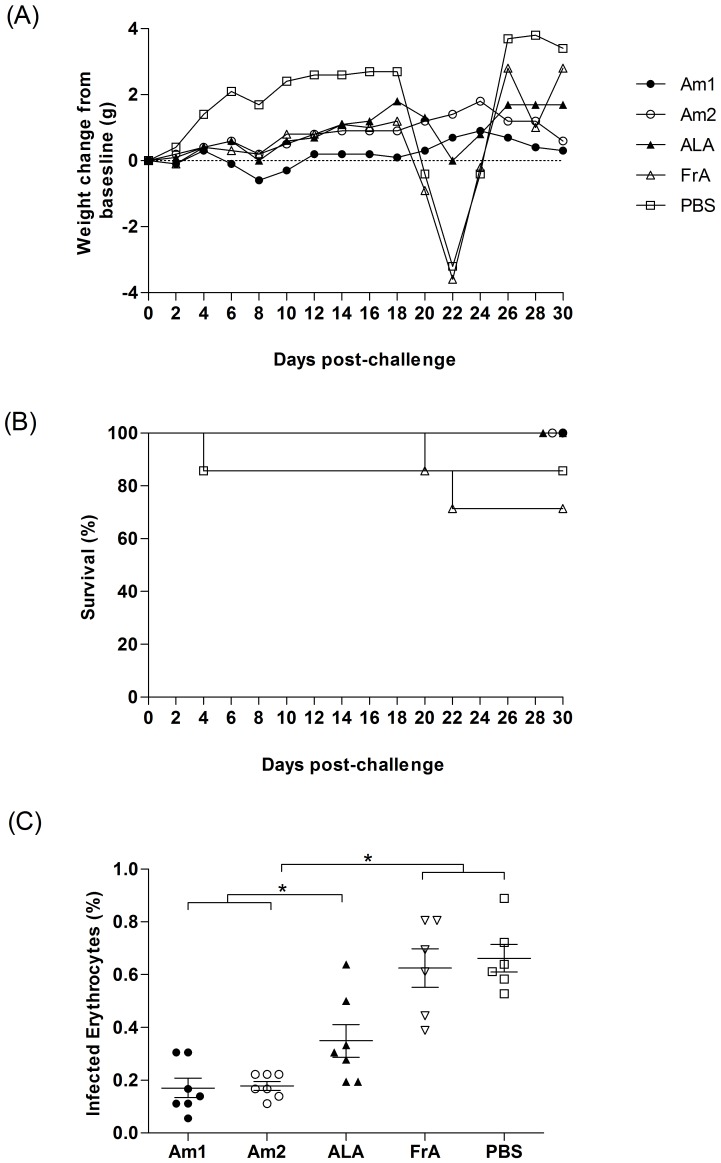
Survival proportions, body weights and infected erythrocytes of mice immunized with synthetic peptides in *Anaplasma marginale* challenge assay. FrA and PBS groups mice showed the highest body weight losses from 18th to 22th day after challenge (A), but the survival percentage of these groups where not significantly different among groups (B). Negative controls included non-immunized and unchallenged mice, which have shown no alteration in weight in comparison to the baseline and in mortality (data not shown). Infected erythrocytes for all groups upon challenge with *A. marginale* are represented as percentage (C) and its infected rate reduction relative to PBS group (D).

Prior to vaccination, we have demonstrated that peptides showed no toxicity to murine macrophage cells, ensuring that any observed effects were not due to peptides toxicity. The body weight was evaluated as a clinical measure of the critical status of the different groups (Figure 2A) and only the non-vaccinated PBS group showed a weight gain, revealing significant weight changes from a baseline when compared to Am1-vaccinated mice (p<0.013). Interestingly, PBS and FrA groups presented considerable weight losses from 18th to 22th days after challenge (Figure 2A). The survival rates of mice immunized with Am1, Am2 and ALA were 100%, while PBS and FrA groups showed lower survival rates (71.4% and 85.7%, respectively) (Figure 2B), although these results were not significantly different. The Am1 and Am2 immunization lead to a barely detectable rickettsemia, since the infected erythrocytes ranged from 0.1% to 0.3%. On other hand, the range of rickettsemia in ALA, PBS and FrA groups were extended from 0.2% to 0.9% (Figure 2C). Then, the relative infection rate reduction of both peptides were 73% and 74%, respectively, while ALA-immunized animals presented 47% of reduction of infected erythrocytes at day 30 after challenge (Figure 2D).

### Differential Response of Specific IgG Isotypes during Immunization and after Challenge

**Figure 3:**
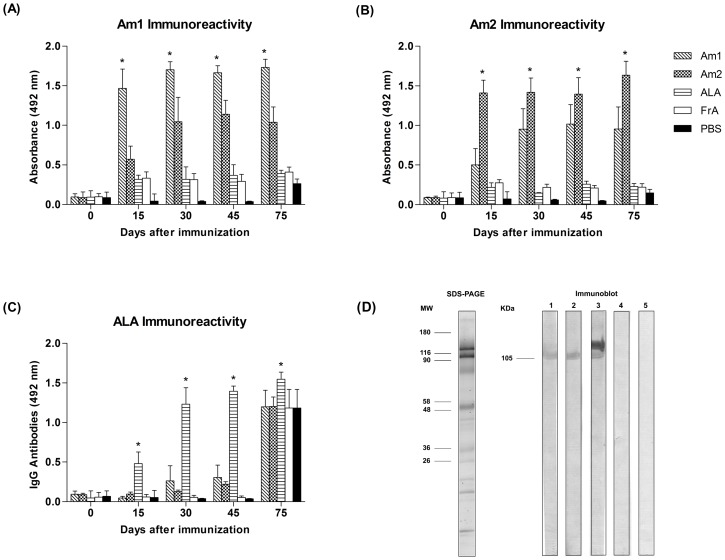
Profile of IgG antibody formation during the whole stage of experiment. Total IgG antibody response of BALB/C mice immunized subcutaneously three times with Am1, Am2, *Anaplasma* Lysate Antigen (ALA), adjuvant control (FrA) or PBS (infection control) against Am1 (A), Am2 (B) and ALA (C), determined by ELISA. Mice were challenged with 3×10^5^ rickettsia after the 45^th^ day. Blood samples were collected at 0, 15, 30, 45 and 75 days after immunization. *Statistically significant differences (p<0.001). (D) ALA SDS-PAGE 12% stained with colloidal comassie and western immunobloting of ALA detected by sera of immunized mice with Am1 (lane 1), Am2 (lane 2), ALA (lane 3), Freund adjuvant (lane 4) and PBS (lane 5). Molecular weight (MW, kDa) and the immunodominant antigen are indicated.

Specific IgG production to each antigen after immunization protocols is shown in Figure 3. As expected, Am1, Am2 and ALA-immunized mice group presented the highest reactivity against Am1 (Figure 3A), Am2 (Figure 3B) and ALA (Figure 3C), respectively, during immunization (day 15 to 45) and after challenge (day 75), in comparison to other groups (p<0.0001). Sera did not show reactivity to BSA used in the control reaction. Immunoblot results also showed a distinct IgG antibody reactivity profile exhibited by sera from immunized animals, recognizing an ALA antigenic band of 105 kDa, which was revealed by sera of Am1, Am2 and ALA groups, but not detected in FrA and PBS groups (Figure 3D). Sera from the ALA group also reacted to an unspecific protein band of >116 kDa.

**Figure 4:**
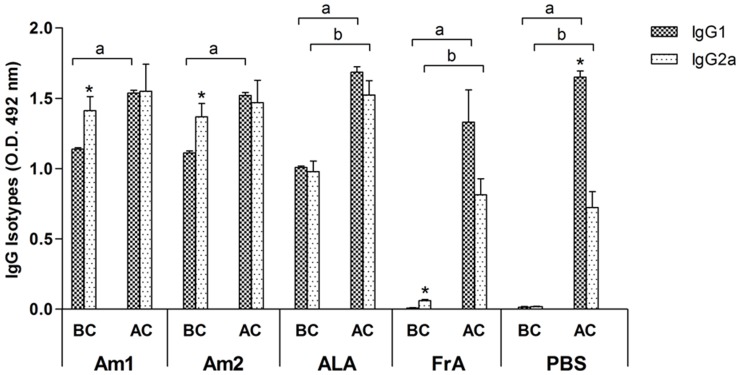
Serum IgG isotypes levels and profile. Specific IgG1 and IgG2a response were analyzed in the serum of mice before challenge (BC) and 30 days after challenge (AC). *Statistically significant differences between IgG1 and IgG2a; ^a^statistically significant differences between IgG1 BC and AC; ^b^statistically significant differences between IgG2a, BC and AC (p<0.05).

Antibody isotype responses were compared before and after parasite challenge in all experimental groups (Figure 4). Levels of IgG2a were significantly higher than IgG1 in mice immunized with Am1 or Am2 (p<0.05), except for the FrA control that showed a significant and opposite response (p<0.05), a profile that was followed by the PBS group, although not significantly different. In the overall response, immunization with *Anaplasma* antigens’ immunizations presented two-fold higher levels of IgG2a than IgG1 in comparison to controls (FrA and PBS), but they were not different within antigenic treatments (p>0.05).

### Am1 and Am2 Differentially Regulated the Transcription of Pivotal Anti- and Pro-inflammatory Cytokines at Immunization and Challenge Periods

**Figure 5:**
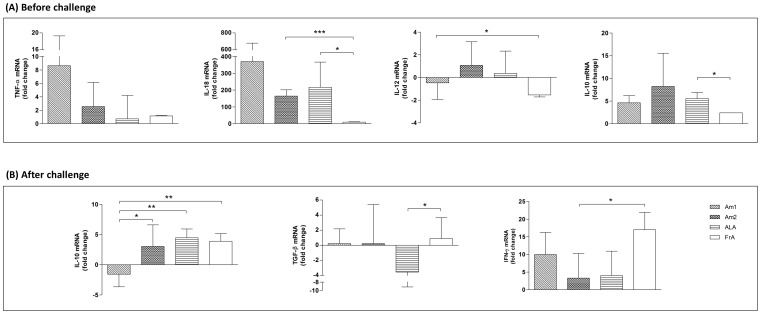
Effects of immunization on cytokine expression of mice splenocytes. The Cytokine expression was quantified before challenge (A) and after *A. marginale* challenge (B) by real-time PCR with focus on mRNA expression of IL-12, IL-18, IFN-γ, TNF-α, IL-10 and TGF-β. Results are presented as the mean ± SE of the fold-change of mRNA in peptides, ALA or FrA immunized mice (n = 3, A; n = 7, B) compared to negative control (PBS-immunized mice). Animals were challenged with 3×10^5^ rickettsia after 45th day. *Statistically significant differences (p<0.05); **p<0.01; ***p<0.0001.

Before challenge (Figure 5A), the ALA-immunized animals demonstrated higher relative expression of IL-10 and IL-18 in the splenocytes, when compared to the PBS control group. Interestingly, mice immunized with the Am1 peptide presented higher splenic expression of pro-inflammatory IL-12, IL-18 and TNF-α, compared to ALA and control groups. On the other hand, the Am2 peptide presented a trend for the increment of IL-10 and a significant up-regulation IL-18 in relation to the control groups (Figure 5A).

After challenge (Figure 5B), ALA group sustained the IL-10 expression, while demonstrated a moderate increase of IFN-γ and decrease of TGF-β. Similarly, the Am2 peptide group presented higher IL-10 and lower IFN-γ, when compared to Am1-immunized mice, but maintained the basal level of TGF-β. In contrast, the Am1 peptide group showed the IL-10 down-regulation, while stimulated more than 2-fold relative expression of IFN-γ and basal level of TGF-β, compared to ALA-immunized mice (Figure 5B).

## Discussion

Bovine anaplasmosis vaccines capable of protecting the cattle and inhibit the tick vector capacity are still urgently needed; however, vaccines under evaluation still rely on bovine animals for tests, and no other animal model has proven to be useful, imposing great difficulty to the improvement of preventive measures against this pathogen. In this study, we have successfully infected BALB/c mice with *A. marginale*, similar to what have been described for Human granulocytic anaplasmosis [26], and this mouse model of anaplasmosis proved to be a valuable tool to evaluate novel immunogens prior to bovine challenges.

The MSP1a protein has been shown to be involved in adhesion, infection and tick transmission of *A. marginale*, and is associated with a protective humoral immune response in cattle [8], [14], [27]. It contains a variable number of tandemly repeated peptides in the amino-terminal region that are extracellularly exposed for interaction with host cell receptors [10], [28], [29], in which a conserved neutralization-sensitive B-cell epitope was previously characterized as the motif (Q/E)ASTSS [16], [17], and later on was identified as a full 28-amino acid repetitive sequence with variations in seven residues [28]. However, we have demonstrated through Phage Display technology that the immunodominant epitope, STSSxL, is a critical motif for antibody recognition in cattle, and appears to play a predominant role in dictating the formation of the antigen-antibody complex [21], and because it reacts with close to 100% of infected cattle sera, we hypothesized that it should raise similar immune response and protection induced by the MSP1 protein complex, as shown previously [8], [14], [27].

Therefore, the immunodominance of epitopes seems to be a key factor limiting the adaptive immunity [20] and this investigation was an opportunity to provide evidences that a core epitope-based vaccine approach is possible, especially because most of the immunization protocols have used whole recombinant MSPs [3]–[7] or membrane proteins fractions [14], [30] with significant protection against challenges, as shown by decreased bacteremia, and significant titers against polypeptides, but with variable efficacy.

The present study demonstrated, for the first time, that mice immunized with critical motifs of the MSP1a functional epitope protected mice against *A. marginale* challenge. This fact was evidenced by a significant decrease in ricketsemia in immunized mice with the synthetic peptides. Moreover, specific antibodies from immunized mice sera with Am1, Am2 and ALA antigens have successfully recognized, as demonstrated by western blotting, an antigenic protein with similar molecular weight to MSP1a.

Serological responses after vaccination showed a considerably higher immunogenicity for synthetic peptides and ALA immunized groups in comparison to controls groups, as demonstrated by high levels of specific IgG. The same pattern has been reported elsewhere with a murine model of human granulocytic ehrlichiosis, in which the antibody response reduced the level of rickettsemia, although it did not confer complete protection against challenge [31].

In addition, mice immunized with Am1 and Am2 exhibited a predominance of IgG2a response, after immunization, and IgG2a levels continued at high levels even after challenge, which is corroborated by a report elsewhere show that complete protection against rickettsemia is achieved with the development of an IgG2-specific response prior to challenge [18]. Moreover, the presence of the IgG2 isotype has been considered as an evidence of a Th1-type immune response [32]. The predominantly elevated production of IgG1 elicited in all antigen groups after challenge (Am1, Am2 and ALA), including the FrA and PBS control group, may be explained due to antigen association with Freund’s adjuvant, or bacteria alone in the PBS control, preferentially stimulate the Th2 response, and may vigorously suppress Th1 responses [33], especially in the PBS control group, which showed significant reduction of the IgG2a response.

Antibodies against MSP1s act as opsonins, facilitating phagocytosis and elimination of *A. marginale* [34], besides inhibits erythrocyte invasion by rickettsias [31]. Interestingly, CD4+ T-cell responses detected only against MSP1a [15] are critical for activated macrophages to secrete nitric oxide [35], [36]. This is consistent with the hypothesis that a strong cellular response characterized by IFN-γ and IgG2 production is important for protective immunity of anaplasmosis [18], [37]. However, IFN-γ may play a crucial role in the clearance of the organism, but also may be a major determinant of histopathology-associated lesions, when it is not counterbalanced with appropriate anti-inflammatory response [38].

In order to verify the effect of synthetic peptides in inducing specific response in the mouse immune system, we have analyzed the expression of inflammatory cytokines involved in the early response, such as IL-10, IL-12, IL-18 and TNF-α, and involved in the post-challenge stage as IFN-γ and TGF-β, due to previous reports that demonstrate the connection of these elements with the pathogenesis and protection in related diseases [38]–[44]. We demonstrated that immunizations with Am1 peptide, induced higher expression levels of IL-12, IL-18, and TNF-α. These data has relevant significance, since IL-12 has multiple biological functions, including differentiation and maintenance of naive CD4+ T cells to Th1 cells and activation of NK cells to produce IFN-γ and other Th1 cytokines, thus it bridges innate and adaptive immunity. In addition, IL-12 was found to have synergistic effects with IL-18 in developing Th1 cells, and IL-12 and IL-18 reciprocally upregulate each other’s receptors [45]. IL-18 can also act directly on effector and memory T cells by inducing migration [46], proliferation, and IFN-γ secretion even in the absence of antigens [47], [48]. Additionally, a previous report that demonstrated secretion of IL-18 is previous to IFN-γ production played a pivotal role in *A. phagocytophilum* clearance [49].

Upon challenge, the mice immunized with Am1 peptide revealed to be in line with a Th1 response, showing an up-regulation of IFN-γ and down-regulation of IL-10, which is compatible with an expected clearance of *A. marginale*. In vitro and in vivo models provide strong evidence that IFN-γ protects against infections by obligate intracellular bacteria [50], which includes anaplasmosis as well [18]. However, excessive inflammation may charge its tolls, as cellular activation induced by IFN-γ may potentially damage host tissues, while the anti-inflammatory effect of IL-10 may limit host-mediated tissue injury by down-regulating IFN-γ or other pro-inflammatory cytokines [39], [41]. Then, in contrast to Am1 peptide, the post-challenge response in Am2-immunized animals revealed a different profile against this whole epitope, leaning to up-regulation of IL-10 and a weak up-regulation of IFN-γ.

The mice model presents limitations for *A. marginale* growth and establishment; however, we have successfully demonstrated the deleterious effect of the parasite infection in non-immunized animals (FrA and PBS). As a comparison, the infection in Balb/c mice induced bacteremia levels 98% smaller than those recorded in bovine erythrocytes [51], but it may still be useful model for infection studies in this disease. But, most importantly, the two peptides protected the animals by showing a significant reduction in circulating *Anaplasma* (75%). The high protection rates observed in vaccinated mice with Am1, Am2 or ALA could be associated with an effective humoral immune response characterized by high levels of total IgG, IgG1 and IgG2a response and a protective cellular immune response with an adequate balance of pro-inﬂammatory and regulatory cytokines (IL-12, IL-18, IFN-γ/IL-10 ratio).

Interestingly, although both synthetic peptides share the same critical motif of the MSP1a epitope and presented similar IgG response, they have mediated significant differences in cytokines’ expression profiles before and after challenge, which may be due to the conformational structures of their specific sequences. Although the Am2 peptide represents the full epitope sequence, its 3D structure [21] may be misrepresented in the peptide design, probably masking part of the true core motif that is represented in the Am1 peptide with three repetitive motifs. The simpler structure of the Am1 may directly present the core motif, and this is probably the reason of a higher IFN-γ response and lower IL-10 production observed for this specific peptide. However, it is difficult to speculate which of the peptides will have a better performance in bovine challenges, because a balanced response may be necessary for a correct clearance of the pathogen.

In conclusion, we have demonstrated that the two synthetic peptides obtained from critical motifs within the MSP1a functional epitope were able to induce humoral and cellular immune responses in mice associated with the upregulation of pro-inflammatory cytokines. This work demonstrates that epitope-based vaccines are possible, and suggests that these novel immunogens may induce protective immunity against bovine anaplasmosis, and further tests should be performed in bovine challenges aiming bacterial clearance and diminished pathogenesis.
